# γ-Glutamyltransferase, but not markers of hepatic fibrosis, is associated with cardiovascular disease in older people with type 2 diabetes mellitus: the Edinburgh Type 2 Diabetes Study

**DOI:** 10.1007/s00125-015-3575-y

**Published:** 2015-03-29

**Authors:** Joanne R. Morling, Jonathan A. Fallowfield, Rachel M. Williamson, Christine M. Robertson, Stephen Glancy, Indra N. Guha, Mark W. J. Strachan, Jackie F. Price

**Affiliations:** 1 Centre for Population Health Sciences, University of Edinburgh, Old Medical Buildings, Teviot Place, Edinburgh, EH8 9AG UK; 2 Queen’s Medical Research Institute, University of Edinburgh, Edinburgh, UK; 3 Department of Metabolic Medicine, Western General Hospital, Edinburgh, UK; 4 Department of Radiology, Western General Hospital, Edinburgh, UK; 5 Digestive Diseases Centre, University of Nottingham, Nottingham, UK

**Keywords:** Cardiovascular diseases, Community-based, Epidemiology, Fatty liver, γ-Glutamyltransferase, Type 2 diabetes mellitus

## Abstract

**Aims/hypothesis:**

We examined the association of prevalent and incident cardiovascular disease (CVD) with chronic liver disease in a cohort of community-based people with type 2 diabetes, in order to clarify the relationship between these two important conditions.

**Methods:**

1,066 participants with type 2 diabetes aged 60–75 years underwent assessment of a range of liver injury markers (non-specific injury, steatosis, steatohepatitis, fibrosis, portal hypertension). Individuals were followed up for incident cardiovascular events.

**Results:**

At baseline there were 370/1,033 patients with prevalent CVD, including 317/1,033 with coronary artery disease (CAD). After a mean follow-up of 4.4 years there were 44/663 incident CVD events, including 27/663 CAD events. There were 30/82 CVD-related deaths. Risk of dying from or developing CVD was no higher in participants with steatosis than in those without (HR 0.90; 95% CI 0.40, 2.00; *p* > 0.05). The only notable relationship was with γ-glutamyltransferase (GGT) (incident CVD: adjusted HR for doubling GGT 1.24 [95% CI 0.97, 1.59] *p* = 0.086; incident CAD: adjusted HR 1.33 [95% CI 1.00, 1.78] *p* = 0.053), suggesting that in our study population, chronic liver disease may have little effect on the development of, or mortality from, CVD.

**Conclusions/interpretation:**

An independent association between GGT and CVD warrants further exploration as a potentially useful addition to current cardiovascular risk prediction models in diabetes. However, overall findings failed to suggest that there is a clinical or pathophysiological association between chronic liver disease and CVD in elderly people with type 2 diabetes.

**Electronic supplementary material:**

The online version of this article (doi:10.1007/s00125-015-3575-y) contains peer-reviewed but unedited supplementary material, which is available to authorised users.

## Introduction

Reports of higher cardiovascular mortality rates in people from the general population with non-alcoholic fatty liver disease (NAFLD) [1, 2] raise the possibility that there may be a pathophysiological relationship between NAFLD and the development of cardiovascular disease (CVD). In people with type 2 diabetes, such a relationship could help to explain the higher prevalences of both conditions. However, the association between CVD and NAFLD has not been well researched in diabetic populations, such that the true relationship between these two important conditions remains uncertain. Epidemiological knowledge of the relationship between NAFLD and CVD in diabetes is particularly limited: current studies are restricted to ultrasound scan-detected NAFLD and the secondary care end of the diabetes spectrum [3, 4]. We therefore aimed to determine the association of CVD with a range of biomarkers of chronic liver injury in a large cohort representative of the full spectrum of elderly people with type 2 diabetes.

Biologically, an association between NAFLD and CVD is plausible. Many of the pathogenic factors proposed for NAFLD and atherosclerosis are shared (e.g. insulin resistance, dyslipidaemia, systemic inflammation) and are closely linked to type 2 diabetes. The concept of the liver–vessel axis hypothesis [5] could also explain the biological mechanisms linking the liver directly to the accelerated atherosclerosis proposed in NAFLD. There is evidence indicating that a consequence of advanced NAFLD (non-alcoholic steatohepatitis [NASH]) includes enhanced atherosclerosis via further insulin resistance leading to atherogenic hyperlipidaemia (low HDL-cholesterol, high triacylglycerol and high LDL-cholesterol levels) and systemic inflammation through pro-inflammatory and pro-atherogenic factors (IL-6, TNF-α, nuclear factor kappa-light-chain-enhancer of activated B cells [6, 7]).

One of the challenges in exploring the association between CVD and NAFLD or chronic liver disease in general in human epidemiological studies is the lack of validated methods to diagnose the various stages of chronic liver disease using non-invasive tests which can be ethically applied to large groups of people who are mostly asymptomatic in terms of liver disease. Attempts to categorise people as ‘diseased’ or ‘not diseased’ based on findings of such non-invasive tests in an epidemiological setting are likely to lead to considerable bias. Therefore, we chose to explore the direct association of a wide range of different liver injury biomarkers with CVD rather than attempt to categorise chronic liver disease based on what would be arbitrary cut-points. We examined the association of prevalent and incident CVD with an array of biomarkers, including those measuring non-specific liver injury (plasma liver enzymes), steatosis (ultrasound), steatohepatitis (cytokeratin-18 [CK18] [8]), surrogate of advanced portal hypertension (platelet count), and liver fibrosis (aspartate to platelet ratio index [APRI] [9], aspartate aminotransferase [AST] to alanine aminotransferase [ALT] ratio, fibrosis-4 score [FIB4] [10], enhanced liver fibrosis panel [ELF] [11] and NAFLD fibrosis score [NFS] [12]).

## Methods

### The Edinburgh type 2 Diabetes study

**Fig. 1:**
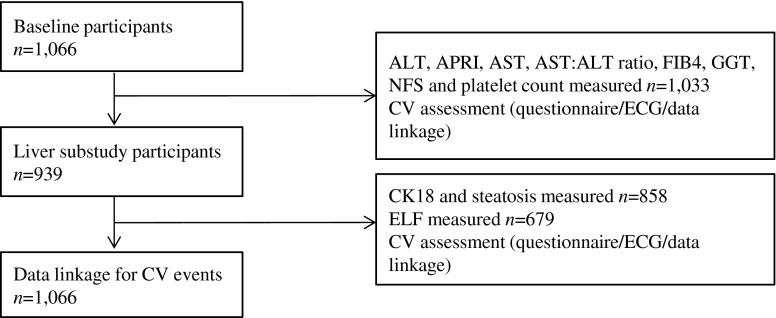
Participant flowchart

Full methods of the Edinburgh Type 2 Diabetes Study (ET2DS) have been published elsewhere [13]. Patients with type 2 diabetes aged 60–75 years at baseline were selected at random from the Lothian Diabetes Register, a comprehensive register of patients with diabetes living in Lothian, Scotland, UK. Baseline attendees (*n* = 1,066) have previously been shown to be representative of all those randomly selected to participate (*n* = 5,454), and therefore representative of the target population of older people with type 2 diabetes living in the general population [14]. The liver assessment clinic was attended by 939 participants at year 1 (Fig. 1).

### Clinical examination

Research clinics were held at the Wellcome Trust Clinical Research Facility, Western General Hospital, Edinburgh, UK, at baseline, year 1 and at follow-up and have been described previously [13, 15]. Briefly, attendees underwent fasting venous blood sampling for measurement of plasma liver enzymes (including ALT, AST and γ-glutamyltransferase [GGT]) and platelets; height and weight recording; blood pressure measurement; and a self-administered questionnaire including standard questions on current medications (including diabetes treatment, defined as diet-controlled, oral antihyperglycaemic agent only or insulin ± oral antihyperglycaemic agent), alcohol consumption, smoking (categorised as ever or never), history of liver disease and CVD, as well as the Edinburgh Claudication and WHO chest pain questionnaires. A 12-lead ECG was also recorded, using recognised standard operating procedures and a MAC 1200 resting ECG analysis system (GE Medical Systems, Milwaukee, Wisconsin, USA), and coded using The Minnesota Code manual [16]. Imaging included abdominal ultrasound scan. Average alcohol intake per week over the previous year and a history of alcohol excess were determined by questionnaire using questions adapted from the Alcohol Use Disorders Identification Test Consumption screening tool. Alcohol excess was defined as >14 units/week in women and >21 units/week in men [17] or self-reported history of an alcohol problem.

NAFLD was defined as the presence of hepatic steatosis on ultrasound scan, without alcohol excess or use of hepatotoxic medication, and a negative liver screen [18].

Alcohol excess was as defined above. Hepatotoxic medication use was defined as the use of non-topical glucocorticoids (isoniazid, methotrexate, amiodarone or tamoxifen) for >2 weeks within the 6 months prior to ultrasound scan. A positive liver screening included any of positive autoantibodies (any of anti-nuclear antibody, anti-smooth muscle antibody, anti-mitochondrial antibody), ferritin >2,247 pmol/l, α-fetoprotein >6 μg/l, or positive hepatitis B or C serology. Clinically significant positive immunology titres were defined as anti-smooth muscle antibody titre >1:160 or anti-mitochondrial antibody titre >1:40 [19].

### Biomarkers of chronic liver injury

Biomarkers of liver injury were categorised and defined as: non-specific liver injury (liver enzyme levels: AST, ALT, GGT), steatosis (ultrasound scan), steatohepatitis (CK18), liver fibrosis (APRI, AST:ALT ratio, ELF, FIB4 and NFS) and advanced portal hypertension (platelet count).

Plasma liver enzymes, APRI, AST:ALT ratio, FIB4, NFS and platelet count were measured at baseline. CK18 and ELF were measured at year 1. All patients underwent a liver ultrasound scan at the 1 year visit. Sonographic grading of hepatic steatosis was performed using standard criteria, as described previously, following validation against proton magnetic resonance spectroscopy [20].

ALT, AST and GGT were analysed using a Vitros Fusion chemistry system (Ortho Clinical Diagnostics, High Wycombe, UK) at the Western General Hospital, Edinburgh, UK. APRI [9], FIB4 [10] and NFS [12] were calculated as in the original publications. AST:ALT ratio was calculated as AST (U/l)/ALT (U/l). CK18 and ELF tests were undertaken on serum samples taken at the time of the liver ultrasound scan and subsequently stored at −80°C. CK18 was measured using the M30-Apoptosense ELISA (Peviva, Stockholm, Sweden) at the Biomedical Research Unit laboratory, University of Nottingham, UK. ELF scores were derived from the serum hyaluronic acid (HA), aminoterminal peptide of procollagen III (P3NP) and tissue inhibitor of metalloproteinases-1 (TIMP-1) equation as in the original publication [11] and measured using the ADVIA Centaur immunoassay system (Siemens Healthcare Diagnostics, New York, NY, USA) at the iQur laboratory, London, UK.

Given that biomarkers of fibrosis (e.g. ELF) could potentially be influenced by the presence of arthropathies [21] and renal disease, the presence of joint diseases (osteoarthritis, rheumatoid arthritis and others) was actively sought through self-administered questionnaire. Estimated glomerular filtration rate (eGFR) was measured at the time of clinic attendance and analysed using a Vitros Fusion chemistry system (Ortho Clinical Diagnostics) at the Western General Hospital, Edinburgh, UK.

### Identifying CVD

Information on cardiovascular events at baseline and at follow-up clinics was collected from multiple sources including patient- and/or general practitioner-completed questionnaires, 12-lead ECG, and linkage to hospital discharge and death certification data. Data linkage was undertaken, via the National Health Service National Services Scotland, to Scottish Morbidity Record (SMR01) general and acute inpatient discharge records using ICD-10 (www.who.int/classifications/icd/en/) (and related ICD-9 [www.icd9data.com/2007/Volume1]) codes and to Office for Population Censuses and Surveys (OPCS) version 4 codes for cardiovascular interventions. A fatal or non-fatal cardiovascular event was recorded if predetermined criteria based on the multiple data sources were met.

#### Myocardial infarction

(1) ICD-10 code for myocardial infarction (MI) on discharge/death record, plus either self-report of a doctor diagnosis of MI, positive WHO chest pain questionnaire for MI, report of MI on general practitioner questionnaire or new ECG codes for MI; or (2) clinical criteria for MI met following scrutiny of clinical notes.

#### Angina

(1) ICD-10 code for angina as primary diagnosis on discharge record; or (2) at least two of (a) self-report of a doctor diagnosis of angina or of starting angina medication, (b) ECG codes for ischaemia, and (c) positive WHO chest pain questionnaire; or (3) clinical diagnosis of angina on scrutiny of hospital notes.

#### Stroke

(1) ICD-10 code for stroke as discharge/death record; or (2) clinical criteria for stroke met on scrutiny of clinical notes in individuals with either self-report of stroke or with non-primary ICD-10 hospital discharge/death code for stroke.

#### Transient ischaemic attack

(1) ICD-10 code for transient ischaemic attack (TIA) on discharge record; or (2) clinical criteria for TIA met on scrutiny of clinical notes in individuals with either self-report of stroke or with non-primary ICD-10 hospital discharge code for stroke or TIA.

#### Coronary intervention

OPCS-4 code for coronary intervention on discharge record.

#### Intermittent claudication

(1) ICD-10 code for intermittent claudication on discharge record; or (2) clinical criteria for intermittent claudication met on scrutiny of clinical notes in individuals with either self-report of intermittent claudication or positive Edinburgh Claudication Questionnaire.

#### Peripheral vascular intervention

OPCS-4 code for peripheral vascular intervention on discharge record.

#### Carotid endarterectomy

OPCS-4 code for carotid endarterectomy on discharge record.

Prevalent CVD at baseline (for ALT, AST, GGT, AST:ALT ratio, APRI, FIB4, NFS and platelets) or year 1 (for steatosis, CK18, ELF) was defined as any of MI, angina, coronary intervention, intermittent claudication, peripheral vascular intervention, stroke, TIA or carotid endarterectomy at any time prior to this point. Prevalent coronary artery disease (CAD) at baseline/year 1 was defined as any of MI, angina or coronary intervention at any time.

Incident CVD was defined as any of MI, angina, coronary intervention, intermittent claudication, peripheral vascular intervention, stroke, TIA or carotid endarterectomy occurring between baseline/year 1 and end of August 2011, for both non-fatal and fatal events, in those patients without prevalent CVD at baseline. Incident CAD was defined as any of MI, angina or coronary intervention occurring between baseline/year 1 and end of August 2011, for non-fatal and fatal events, in those patients without prevalent CAD at baseline.

### Data analysis

The primary outcome measures were prevalent cardiovascular events and incident cardiovascular events. The secondary outcome measures were prevalent and incident CAD events. Fatal and non-fatal events were combined for analysis.

Data were assessed for normality and where necessary non-normal variables (APRI, CK18 and GGT) were transformed on the log_2_ scale.

The follow-up time for each individual for incident disease was from the date of the baseline/liver substudy research clinic attendance until the first of: cardiovascular event, death or end of August 2011.

Analysis was undertaken using a listwise approach for three scenarios—measurements taken at baseline (ALT, APRI, AST, AST:ALT ratio, FIB4, GGT, NFS and platelets), measurements taken at the initial liver substudy clinic (CK18 and steatosis on ultrasound scan) and ELF.

Univariate analysis with normal continuous variables was carried out using Student’s *t* test (ALT, AST, AST:ALT ratio, ELF FIB4, NFS and platelets), non-normal continuous variables (APRI, CK18 and GGT) using the Mann–Whitney *U* test, and categorical variables (steatosis) using the *χ* ^2^ test, examining for both the presence of prevalent and incident CVD and CAD.

Logistic regression for the association with prevalent CVD and CAD, and Cox proportional hazards regression for the association with incident CVD and CAD, were undertaken for all markers of liver injury. Both were performed unadjusted, adjusted for age and sex, and additionally adjusted for age, sex, duration of diabetes, treatment of diabetes, lipid-lowering drugs, blood pressure-lowering drugs, deprivation (Scottish Index of Multiple Deprivation quintile), smoking status, excess alcohol consumption, BMI, systolic blood pressure (sBP), diastolic blood pressure (dBP), HbA_1c_, HDL-cholesterol, total cholesterol and eGFR. Analysis of prevalent disease was undertaken for all participants; analysis of incident disease was undertaken for participants free of CVD at baseline.

Sensitivity analyses of the incident cardiovascular events were undertaken: (1) for participants with NAFLD (defined as the presence of hepatic steatosis on ultrasound scan without alcohol excess or use of hepatotoxic medication and a negative liver screen); and (2) following inclusion of all participants and adjusted for prevalent CVD at baseline.

Data were analysed using SPSS version 19.0 (SPSS, Chicago, IL, USA).

Ethics approval was obtained from the Lothian Research Ethics Committee and all participants gave written informed consent.

## Results

### Patient characteristics

The baseline research clinic was attended by 1,066 patients, 939 (88%) of whom returned for the liver assessment at 1 year. Figure 1 shows the participant flow. There were no significant differences between attenders at baseline and attenders at the liver assessment (reported previously [22]); participant characteristics are described in Table 1.

**Table 1:** Characteristics of all ET2DS participants, those undergoing CK18 and steatosis assessment and subgroups with ELF measurements

Characteristic	All participants (*n* = 1,033)	CK18 and steatosis participants (*n* = 858)	ELF participants (*n* = 679)
Age, years	67.9 (4.2)	67.9 (4.2)	67.8 (4.2)
Sex, % male	51.2 (530)	53.8 (462)	52.6 (357)
Duration of diabetes, years	6.0 (3.0–11.0)	6.0 (3.0–11.0)	6.0 (3.0–10.0)
HbA_1c_, %	7.39 (1.1)	7.38 (1.1)	7.36 (1.1)
HbA_1c_, mmol/mol	57.0 (12.1)	57.2 (12.3)	57.0 (11.9)
Fasting glucose, mmol/l	7.54 (2.1)	7.48 (2.0)	7.49 (2.0)
Diet-controlled, % yes	19.8 (197)	19.3 (161)	19.2 (127)
Oral antihyperglycaemic agent use, % yes	63.0 (628)	64.8 (541)	65.4 (432)
Insulin therapy, % yes	17.3 (172)	15.9 (133)	15.4 (102)
BMI, kg/m^2^	31.3 (5.6)	31.2 (5.7)	31.2 (5.7)
Waist circumference, cm	106.7 (12.7)	106.6 (12.8)	106.5 (12.7)
Serum cholesterol, mmol/l	4.30 (0.9)	4.31 (0.9)	4.33 (0.9)
sBP, mmHg	133.2 (16.4)	133.3 (16.1)	133.5 (16.3)
dBP, mmHg	69.1 (9.0)	69.3 (8.9)	69.4 (8.9)
Alcohol excess^a^, % yes	8.1 (84)	7.6 (65)	8.4 (57)
Ever smoked, % yes	60.7 (527)	59.6 (455)	60.0 (366)

Values are mean (SD), median (interquartile range) or proportion (*n*)

All variables were measured concurrently at year 1 examination of the ET2DS, except for BMI and waist circumference, which were measured at baseline

^a^Defined as women >14 units/week, men >21 units/week or patient disclosed history of a current or prior alcohol problem

Full data from baseline were available for 1,033 participants. From the 1 year liver assessment, steatosis and CK18 data were available for 858 participants. ELF data were available on a random subgroup of 679 participants; there were no significant differences between participants with and without available ELF scores (Table 1).

### Prevalent CVD

At baseline there were 370/1,033 (35.8%) patients with prevalent CVD and 317/1,033 (30.7%) with prevalent CAD. A significantly higher proportion of those with CVD and CAD were male (both 61.8%, *p* < 0.001) compared with those free of disease. Those with CVD and CAD were older (mean 68.4 vs 67.6 years, *p* = 0.004, and 68.6 vs 67.6 years, *p* < 0.001, respectively) than those without. Results were similar for the 1 year assessment: at baseline there were 303/858 (35.3%) patients with prevalent CVD and 260/858 (30.3%) with prevalent CAD. Again, those with CVD and CAD were significantly more likely to be male and to be older than those without.

There were no significant differences in the distribution of joint disease potentially influencing fibrosis biomarkers between those with and those without CVD (osteoarthritis 22.3% vs 23.8%, *p* = 0.785; rheumatoid arthritis 5.3% vs 3.2%, *p* = 0.173; other joint disease 15.6% vs 12.5%, *p* = 0.440, respectively). Mean eGFR was lower in those with prevalent CVD than in those without (62.1 vs 65.7 ml^−1^ min^−1^ 1.73 m^−2^, *p* < 0.001).

Participants with prevalent CVD had marginally lower ALT (mean 41.9 vs 43.7 U/l, *p* = 0.048) and higher GGT measures (median 20.0 vs 17.0 U/l, *p* < 0.001) compared with those without. Patients with prevalent CAD also had significantly higher GGT values than those without (median 20.0 vs 17.0 U/l, *p* < 0.001), although all median levels were within the normal range. The proportion of participants with steatosis was lower in those with CVD than in those without (CVD 54.1% vs 57.5%, *p* = 0.350; CAD 51.2% vs 58.5%, *p* = 0.051). Full data are given in Table 1 of the electronic supplementary material (ESM).

Multivariable analysis of the relationship between liver markers and prevalent cardiovascular events, adjusting for age, sex, duration of diabetes, treatment of diabetes, lipid-lowering drugs, blood pressure-lowering drugs, deprivation, smoking status, excess alcohol consumption, BMI, systolic blood pressure, HbA_1c_, HDL-cholesterol, total cholesterol and eGFR, is shown in Table 2. GGT was the only liver marker independently associated with prevalent CVD (OR for a doubling of GGT 1.18; 95% CI 1.03, 1.36; *p* = 0.021) or CAD (OR 1.21; 95% CI 1.05, 1.40; *p* = 0.008).

**Table 2:** Multivariable association between liver markers and prevalent cardiovascular events

Liver marker	Model 1	*p* value	Model 2	*p* value	Model 3	*p* value
All CVD						
ALT, U/l	0.99 (0.98, 1.00)	0.079	0.99 (0.98, 1.00)	0.028	0.99 (0.98, 1.00)	0.088
AST, U/l	0.99 (0.98, 1.01)	0.341	0.99 (0.98, 1.01)	0.174	0.99 (0.98, 1.01)	0.385
GGT, log_2_^a^	1.21 (1.07, 1.37)	0.002	1.20 (1.06, 1.35)	0.005	1.18 (1.03, 1.36)	0.021
Steatosis, % yes	0.91 (0.68, 1.22)	0.518	0.96 (0.71, 1.30)	0.774	0.84 (0.60, 1.17)	0.296
CK18, log_2_^a^	1.08 (0.90, 1.30)	0.421	1.09 (0.90, 1.31)	0.405	0.99 (0.81, 1.22)	0.926
APRI, log_2_^a^	0.98 (0.78, 1.23)	0.833	0.85 (0.67, 1.08)	0.189	0.90 (0.70, 1.67)	0.439
AST:ALT ratio	1.34 (0.56, 3.21)	0.509	1.39 (0.56, 3.44)	0.473	1.51 (0.56, 4.07)	0.419
ELF score	1.00 (0.83, 1.21)	0.984	1.01 (0.82, 1.23)	0.964	0.94 (0.74, 1.19)	0.604
FIB4	1.13 (0.90, 1.41)	0.289	1.01 (0.80, 1.28)	0.921	1.03 (0.80, 1.33)	0.801
NFS	1.09 (0.96, 1.24)	0.174	1.07 (0.93, 1.22)	0.350	1.01 (0.85, 1.19)	0.915
Platelets, ×10^9^/l	1.00 (1.00, 1.00)	0.569	1.00 (1.00, 1.00)	0.499	1.00 (1.00, 1.00)	0.734
CAD						
ALT, U/l	0.99 (0.98, 1.00)	0.200	0.99 (0.98, 1.00)	0.124	0.99 (0.98, 1.01)	0.390
AST, U/l	0.99 (0.98, 1.01)	0.383	0.99 (0.98, 1.01)	0.230	1.00 (0.98, 1.01)	0.588
GGT, log_2_^a^	1.22 (1.08, 1.39)	0.002	1.22 (1.07, 1.38)	0.002	1.21 (1.05, 1.40)	0.008
Steatosis, % yes	0.75 (0.55, 1.02)	0.064	0.79 (0.58, 1.08)	0.140	0.66 (0.46, 0.94)	0.019
CK18, log_2_^a^	1.05 (0.86, 1.28)	0.650	1.05 (0.86, 1.28)	0.610	0.96 (0.78, 1.18)	0.707
APRI, log_2_^a^	1.00 (0.79, 1.27)	0.987	0.88 (0.67, 1.13)	0.320	0.95 (0.73, 1.24)	0.720
AST:ALT ratio	1.01 (0.40, 2.53)	0.981	0.93 (0.36, 2.42)	0.887	0.94 (0.33, 2.66)	0.912
ELF score	0.98 (0.80, 1.20)	0.848	0.96 (0.77, 1.19)	0.726	0.88 (0.69, 1.13)	0.324
FIB4	1.20 (0.95, 1.50)	0.123	1.07 (0.84, 1.36)	0.599	1.11 (0.85, 1.43)	0.441
NFS	1.11 (0.97, 1.27)	0.138	1.07 (0.93, 1.23)	0.328	1.03 (0.86, 1.22)	0.765
Platelets, ×10^9^/l	1.00 (1.00, 1.00)	0.386	1.00 (1.00, 1.00)	0.788	1.00 (1.00, 1.00)	0.967

Values are ORs (95% CI)

^a^APRI, CK18 and GGT analysed on the log_2_ scale for linearisation; therefore, ORs relate to a doubling of the marker

Model 1, unadjusted; model 2, adjusted for age and sex; model 3, adjusted for age, sex, duration of diabetes, treatment of diabetes, lipid-lowering drugs, blood pressure-lowering drugs, deprivation (Scottish Index of Multiple Deprivation quintile), smoking status, excess alcohol consumption, BMI, sBP, dBP, HbA_1c_, HDL-cholesterol, total cholesterol and eGFR at baseline

### Incident CVD

There were 663 participants without CVD. After a mean follow-up of 4.4 years from baseline attendance there were 44/663 (6.6%) patients with incident CVD and 27/663 (4.1%) with incident CAD events. A significantly higher proportion of those with incident CVD were male (59.1% vs 44.3%, *p* = 0.061) and they were significantly older (68.9 vs 67.5 years, *p* = 0.024), with no differences in those with incident CAD compared with those without incident CAD. Similar results were obtained for those patients followed up from the 1 year assessment (mean follow-up 3.5 years), with 35/561 (6.2%) incident CVD and 19/561 (3.4%) incident CAD events and with a similar age/sex distribution.

There were 82/1,033 (7.9%) deaths in the follow-up period from baseline, with 30/82 (36.6%) attributable to CVD, of which 20 were attributable to CAD.

Mean (or median) liver injury marker levels were largely similar between participants with and without incident CVD (ESM Table 2) and after multivariable adjustment (Table 3). Only GGT appeared to have some independent association with either incident CVD (HR for a doubling of GGT 1.24; 95% CI 0.97, 1.59; *p* = 0.086) or incident CAD (HR 1.33; 95% CI 1.00, 1.78; *p* = 0.053). None of the individual covariables added to the multivariable model had a major attenuating effect on the HR estimating the GGT–outcome association (ESM Table 3). In further analyses performed on all participants with either a first or subsequent cardiovascular event occurring after baseline (i.e. including those with prevalent CVD at baseline, but with adjustment for prevalent cases), an association between GGT and events was confirmed (ESM Tables 4 and 5). HRs with similar magnitudes were observed with increased statistical significance (*p* < 0.05), likely due to the increase in sample size.

**Table 3:** Multivariable association between liver markers and any incident CVD events

Liver marker	Model 1	*p* value	Model 2	*p* value	Model 3	*p* value
All CVD						
ALT, U/l	1.00 (0.97, 1.02)	0.754	1.00 (0.97, 1.02)	0.836	0.99 (0.97, 1.02)	0.669
AST, U/l	1.01 (0.98, 1.04)	0.526	1.01 (0.98, 1.04)	0.544	1.01 (0.97, 1.04)	0.700
GGT, log_2_^a^	1.25 (0.99, 1.59)	0.062	1.26 (0.99, 1.60)	0.059	1.24 (0.97, 1.59)	0.086
Steatosis, % yes	0.78 (0.36, 1.67)	0.525	0.84 (0.39, 1.80)	0.654	0.90 (0.40, 2.00)	0.787
CK18, log_2_^a^	1.05 (0.64, 1.70)	0.857	1.13 (0.68, 1.85)	0.643	1.02 (0.60, 1.75)	0.931
APRI, log_2_^a^	0.88 (0.505, 1.525)	0.644	0.79 (0.43, 1.46)	0.448	0.76 (0.40, 1.45)	0.408
AST:ALT ratio	3.63 (0.61, 21.61)	0.156	2.85 (0.475, 17.06)	0.252	3.58 (0.53, 28.12)	0.183
ELF score	1.220 (0.91, 1.64)	0.185	1.19 (0.85, 1.66)	0.312	1.15 (0.81, 1.64)	0.443
FIB4	1.01 (0.54, 1.91)	0.966	0.82 (0.40, 1.68)	0.586	0.83 (0.39, 1.76)	0.625
NFS	0.81 (0.58, 1.14)	0.226	0.76 (0.54, 1.06)	0.109	0.78 (0.57, 1.09)	0.143
Platelets, ×10^9^/l	1.00 (1.00, 1.01)	0.162	1.01 (1.00, 1.01)	0.061	1.00 (1.00, 1.01)	0.110
CAD						
ALT, U/l	1.00 (0.98, 1.03)	0.771	1.01 (0.98, 1.04)	0.497	1.01 (0.98, 1.04)	0.611
AST, U/l	1.02 (0.99, 1.05)	0.213	1.03 (0.99, 1.06)	0.135	1.02 (0.99, 1.06)	0.220
GGT, log_2_^a^	1.27 (0..95, 1.69)	0.103	1.31 (9.88, 1.75)	0.060	1.33 (1.00, 1.78)	0.053
Steatosis, % yes	0.82 (0.32, 2.14)	0.688	0.87 (0.33, 2.27)	0.774	0.91 (0.33, 2.53)	0.858
CK18, log_2_^a^	1.07 (0.58, 1.99)	0.822	1.10 (0.60, 2.01)	0.748	0.96 (0.49, 1.90)	0.908
APRI, log_2_^a^	1.07 (0.56, 2.06)	0.839	1.15 (0.56, 2.34)	0.709	1.10 (0.52, 2.32)	0.804
AST:ALT ratio	4.36 (0.51, 37.18)	0.178	3.40 (0.37, 31.13)	0.278	4.25 (0.39, 46.73)	0.237
ELF score	1.24 (0.85, 1.80)	0.269	1.15 (0.76, 1.74)	0.508	1.12 (0.69, 1.82)	0.642
FIB4	1.28 (0.64, 2.60)	0.486	1.22 (0.57, 2.64)	0.611	1.25 (0.56, 2.79)	0.583
NFS	0.84 (0.55, 1.28)	0.416	0.81 (0.53, 1.23)	0.323	0.76 (0.51, 1.17)	0.225
Platelets, ×10^9^/l	1.00 (1.00, 1.01)	0.301	1.00 (1.00, 1.01)	0.286	1.00 (1.00, 1.01)	0.297

Values are HRs (95% CI)

^a^APRI, CK18 and GGT analysed on the log_2_ scale for linearisation; therefore, ORs relate to a doubling of the marker

Model 1, unadjusted; model 2, adjusted for age and sex; model 3, adjusted for age, sex, duration of diabetes, treatment of diabetes, lipid-lowering drugs, blood pressure-lowering drugs, deprivation (Scottish Index of Multiple Deprivation quintile), smoking status, excess alcohol consumption, BMI, sBP, dBP, HbA_1c,_ HDL-cholesterol, total cholesterol and eGFR at baseline

When restricted to patients with NAFLD (*n* = 319) there were 38 incident cardiovascular events, with 23 attributable to CAD. Of all the liver injury markers investigated, GGT alone showed an independent association with incident CVD in this subgroup (fully adjusted HR for a doubling of GGT 1.56; 95% CI 1.08, 2.28; *p* = 0.019) (ESM Tables 6 and 7).

## Discussion

In this large-scale epidemiological study, we have shown that raised GGT is independently associated with an increase in both prevalent and incident cardiovascular events in older people with type 2 diabetes. Previous studies, predominantly in younger samples of the general population, have found similar results for this plasma liver enzyme; we have now shown that findings are consistent in a high-risk (diabetic) and older subgroup of the population. Despite the availability of a wide range of other liver injury markers, we found no evidence that markers of hepatic steatosis, steatohepatitis, portal hypertension or fibrosis were associated with higher levels of prevalent or incident CVD, suggesting that liver disease may have little effect on the development of vascular complications in our study population.

A major strength of this study is its representation of the full spectrum of people with type 2 diabetes, not just those attending secondary care or receiving advanced treatment modalities. This population is of particular interest as it may show an accelerated progression of liver disease due to the combined effects of age and metabolic risk factors. Community-based populations of people with type 2 diabetes represent the vast majority of all people with type 2 diabetes and, as such, require special attention given the impact of their longer term care on health service provision.

Our findings are consistent with previous findings of a significant association between GGT and both prevalent and incident CVD in the general population [3, 23–30], contributing to the paucity of literature in diabetic populations. In addition, contrary to previous findings, we found that this association persists into older age [26], independently of a wide range of cardiovascular risk factors. There is a biological plausibility for this relationship: GGT degrades glutathione to glutamate, which via cysteinylglycine is involved in iron reduction, allowing lipoprotein oxidation within atheromatous plaques [31]. What is unclear is whether GGT is a pathogenic factor in atherogenesis or simply a surrogate biomarker of the microinflammatory, plaque-associated inflammatory response. Given that no liver injury markers other than GGT were independently associated with CVD, this strengthens the argument for the GGT association being driven by systemic inflammation as opposed to a direct consequence of chronic liver disease. Whatever the underlying mechanism, our findings indicate that further investigation is warranted into whether or not GGT could add predictive ability to existing vascular risk prediction models in type 2 diabetes [32].

In terms of the association between CVD and other liver injury markers, previous studies are limited and inconclusive. Significant associations between transaminases and both increased and decreased CVD in the general population have been reported [33, 34]. Investigations into the relationship between NAFLD (defined as the presence of hepatic steatosis on ultrasound scan) and cardiovascular events [35, 36], in populations comprised exclusively of patients with type 2 diabetes [3, 4, 37, 38], have reported significant associations between NAFLD and incident CVD (OR 1.53 [3], HR 1.96 [38], after controlling for cardiovascular risk factors), but no association with liver enzymes (including GGT). Although the present study failed to find a similar relationship between sonographic hepatic steatosis and CVD, our cohort differs from diabetic cohorts studied previously, mainly in its broad spectrum of patients with type 2 diabetes. Targher et al used a study population derived exclusively from secondary care diabetes settings (therein limiting generalisability), where the influence of hepatic steatosis may be stronger in the context of more severe diabetes, consistent with other studies looking at more general populations and cardiovascular mortality [37]. Whilst our findings may also be affected by specific cohort effects, the size and follow-up time are comparable to those of several other similar studies [3, 26].

Our finding of a lower prevalence of CVD in people with steatosis could be explained, at least in part, by regression of hepatic steatosis with advancing liver disease [39]; or it may reflect survival bias, in that those with the most severe NAFLD had already died prior to participation in the ET2DS.

In patients with NAFLD, relative concentrations of serum CK18 can discriminate between steatosis and NASH [8]. However, there are no previous studies examining the relationship between CK18 levels and cardiovascular events in either general or diabetic populations. Several previous studies diagnosing NASH using different methods (such as biopsy or elevated ALT levels) showed mixed results for the association with cardiovascular risk (e.g. risk scores, lipid levels). Both Soderberg et al [40] and Ekstedt et al [2] found associations of all-cause and cardiovascular mortality with the presence of biopsy-proven NASH, but no association with steatosis. Conversely, Lazo et al [41] found no association between NASH and cardiovascular mortality in patients diagnosed by ultrasound scan and elevated hepatic enzymes, suggesting that the criteria for NAFLD and NASH classification may have a significant impact on findings.

Data on the relationship between hepatic fibrosis and CVD are also limited. Kim et al found significant associations between the NFS, APRI and FIB4 with cardiovascular mortality in a general population [42]. Our study used all these, as well as the ELF score, an extracellular matrix-related multi-component panel (HA, P3NP and TIMP-1), validated for use in patients with NAFLD [20], and found no relationship.

It should be noted that the utility of different liver injury biomarkers may be determined by the context in which they are used. For example, there is a body of evidence validating non-invasive liver biomarkers for the cross-sectional stratification of liver disease in secondary care and predicting future liver-related clinical outcomes [43, 44]. Results from this study do not suggest that most of the markers investigated would add prognostic value to existing risk scores used to predict cardiovascular endpoints in diabetes [32]. The exception to this is GGT, which is generally not considered useful for stratifying active liver disease, but which may prove beneficial in predicting CVD. Given the results presented here, further investigation into this question in diabetes is warranted.

The strengths and limitations of this study should be acknowledged. The large size, population-based approach, prospective design with intensive investigation for incident cardiovascular events, and wide range of liver biomarkers investigated are key strengths of the current study. The modest follow-up duration is partially offset by the large sample size, resulting in a significant number of person-years at risk, and by the high-risk population under study, which resulted in a high number of incident events. Without a liver biopsy it is not currently possible to accurately identify NAFLD. However, we believe that our comprehensive approach of using ultrasound scan, assessment of alcohol consumption and hepatotoxic medication use, and liver screen will identify the vast majority of patients with NAFLD, potentially missing only those with minimal hepatic steatosis due to regression of steatosis in the advanced stages of the disease process.

In conclusion, our study provides evidence that GGT may independently associate with CVD and that its potential prognostic value for CVD in people with type 2 diabetes would be usefully investigated. However, lack of association between CVD and other markers of liver injury (non-specific injury, steatosis, steatohepatitis, significant portal hypertension, fibrosis) suggests that chronic liver disease per se may not have a major influence on the development of CVD, at least in older diabetic populations.

## Electronic supplementary material

Below is the link to the electronic supplementary material.

ESM Table 1 (PDF 185 kb)

ESM Table 2 (PDF 98 kb)

ESM Table 3 (PDF 70 kb)

ESM Table 4 (PDF 165 kb)

ESM Table 5 (PDF 88 kb)

ESM Table 6 (PDF 162 kb)

ESM Table 7 (PDF 162 kb)

## Abbreviations

ALT: Alanine aminotransferase
APRI: Aspartate to platelet ratio index
AST: Aspartate aminotransferase
CAD: Coronary artery disease
CK18: Cytokeratin-18
CVD: Cardiovascular disease
dBP: Diastolic blood pressure
eGFR: Estimated glomerular filtration rate
ELF: Enhanced Liver Fibrosis panel
ET2DS: Edinburgh Type 2 Diabetes Study
FIB4: Fibrosis-4 score
GGT: γ-Glutamyltransferase
HA: Hyaluronic acid
MI: Myocardial infarction
NAFLD: Non-alcoholic fatty liver disease
NASH: Non-alcoholic steatohepatitis
NFS: NAFLD fibrosis score
OPCS: Office for Population Censuses and Surveys
P3NP: Aminoterminal peptide of procollagen III
sBP: Systolic blood pressure
TIA: Transient ischaemic attack
TIMP-1: Tissue inhibitor of metalloproteinases-1

## References

[1] Calori G, Lattuada G, Ragogna F (2011). Fatty liver index and mortality: the Cremona study in the 15th year of follow-up. Hepatology.

[2] Ekstedt M, Franzen LE, Mathiesen UL (2006). Long-term follow-up of patients with NAFLD and elevated liver enzymes. Hepatology.

[3] Targher G, Bertolini L, Poli F (2005). Nonalcoholic fatty liver disease and risk of future cardiovascular events among type 2 diabetic patients. Diabetes.

[4] Targher G, Bertolini L, Rodella S (2007). Nonalcoholic fatty liver disease is independently associated with an increased incidence of cardiovascular events in type 2 diabetic patients. Diabetes Care.

[5] Loria P, Lonardo A, Targher G (2008). Is liver fat detrimental to vessels? Intersections in the pathogenesis of NAFLD and atherosclerosis. Clin Sci (Colch).

[6] Adiels M, Taskinen M-R, Borén J (2008). Fatty liver, insulin resistance, and dyslipidemia. Curr Diab Rep.

[7] Van Gaal LF, Mertens IL, Christophe E (2006). Mechanisms linking obesity with cardiovascular disease. Nature.

[8] Feldstein AE, Wieckowska A, Lopez AR, Liu Y-C, Zein NN, McCullough AJ (2009). Cytokeratin-18 fragment levels as noninvasive biomarkers for nonalcoholic steatohepatitis: a multicenter validation study. Hepatology.

[9] Wai C-T, Greenson JK, Fontana RJ (2003). A simple noninvasive index can predict both significant fibrosis and cirrhosis in patients with chronic hepatitis C. Hepatology.

[10] Vallet-Pichard A, Mallet V, Nalpas B (2007). FIB-4: an inexpensive and accurate marker of fibrosis in HCV infection. Comparison with liver biopsy and fibrotest. Hepatology.

[11] Guha IN, Parkes J, Roderick P (2008). Noninvasive markers of fibrosis in nonalcoholic fatty liver disease: validating the European liver fibrosis panel and exploring simple markers. Hepatology.

[12] Angulo P, Hui JM, Marchesini G (2007). The NAFLD fibrosis score: a noninvasive system that identifies liver fibrosis in patients with NAFLD. Hepatology.

[13] Price J, Reynolds R, Mitchell R (2008). The Edinburgh Type 2 Diabetes study: study protocol. BMC Endocr Disord.

[14] Marioni RE, Strachan MWJ, Reynolds RM (2010). Association between raised inflammatory markers and cognitive decline in elderly people with type 2 diabetes: the Edinburgh Type 2 Diabetes study. Diabetes.

[15] Morling JR, Strachan MWJ, Hayes PC (2012). Prevalence of abnormal plasma liver enzymes in older people with Type 2 diabetes. Diabet Med.

[16] Prineas RJ, Crow RS, Zhang Z (2010). The Minnesota code manual of electrocardiographic findings.

[17] Royal College of Physicians (2011) Science and Technology Select Committee. Inquiry on alcohol guidelines. Royal College of Physicians, London

[18] Chalasani N, Younossi Z, Lavine JE (2012). The diagnosis and management of non-alcoholic fatty liver disease: practice guideline by the American Association for the Study of Liver Diseases, American College of Gastroenterology, and the American Gastroenterological Association. Hepatology.

[19] Alvarez F, Berg PA, Bianchi FB (1999). International Autoimmune Hepatitis Group Report: review of criteria for diagnosis of autoimmune hepatitis. J Hepatol.

[20] Williamson RM, Perry E, Glancy S (2011). The use of ultrasound to diagnose hepatic steatosis in type 2 diabetes: intra- and interobserver variability and comparison with magnetic resonance spectroscopy. Clin Radiol.

[21] Emlen W, Niebur J, Flanders G, Rutledge J (1996). Measurement of serum hyaluronic acid in patients with rheumatoid arthritis: correlation with disease activity. J Rheumatol.

[22] Williamson RM, Price JF, Glancy S (2011). Prevalence of and risk factors for hepatic steatosis and nonalcoholic fatty liver disease in people with type 2 diabetes: the Edinburgh Type 2 Diabetes Study. Diabetes Care.

[23] Monami M, Bardini G, Lamanna C (2008). Liver enzymes and risk of diabetes and cardiovascular disease: results of the Firenze Bagno a Ripoli (FIBAR) study. Metabolism.

[24] Lee D-H, Silventoinen K, Hu G (2006). Serum gamma-glutamyltransferase predicts non-fatal myocardial infarction and fatal coronary heart disease among 28 838 middle-aged men and women. Eur Heart J.

[25] Lee DS, Evans JC, Robins SJ (2007). Gamma glutamyl transferase and metabolic syndrome, cardiovascular disease, and mortality risk: the Framingham Heart Study. Arterioscler Thromb Vasc Biol.

[26] Lee D-H, Buijsse B, Steffen L, Holtzman J, Luepker R, Jacobs DR (2009). Association between serum gamma-glutamyltransferase and cardiovascular mortality varies by age: the Minnesota Heart Survey. Eur J Cardiovasc Prev Rehabil.

[27] Emdin M, Passino C, Michelassi C, Donato L, Pompella A, Paolicchi A (2009). Additive prognostic value of gamma-glutamyltransferase in coronary artery disease. Int J Cardiol.

[28] Strasak AM, Kelleher CC, Klenk J (2008). Longitudinal change in serum gamma-glutamyltransferase and cardiovascular disease mortality: a prospective population-based study in 76,113 Austrian adults. Arterioscler Thromb Vasc Biol.

[29] Hozawa A, Okamura T, Kadowaki T (2007). γ-Glutamyltransferase predicts cardiovascular death among Japanese women. Atherosclerosis.

[30] Kazemi-Shirazi L, Endler G, Winkler S, Schickbauer T, Wagner O, Marsik C (2007). Gamma glutamyltransferase and long-term survival: is it just the liver?. Clin Chem.

[31] Grundy SM (2007). Gamma-glutamyl transferase: another biomarker for metabolic syndrome and cardiovascular risk. Arterioscler Thromb Vasc Biol.

[32] D’Agostino RB, Vasan RS, Pencina MJ (2008). General cardiovascular risk profile for use in primary care the Framingham Heart Study. Circulation.

[33] Schooling CM, Kelvin EA, Jones HE (2012). Alanine transaminase has opposite associations with death from diabetes and ischemic heart disease in NHANES III. Ann Epidemiol.

[34] Schindhelm RK, Dekker JM, Nijpels G (2007). Alanine aminotransferase predicts coronary heart disease events: a 10-year follow-up of the Hoorn Study. Atherosclerosis.

[35] Stepanova M, Younossi ZM (2012). Independent association between nonalcoholic fatty liver disease and cardiovascular disease in the US population. Clin Gastroenterol Hepatol.

[36] Hamaguchi M, Kojima T, Takeda N (2007). Nonalcoholic fatty liver disease is a novel predictor of cardiovascular disease. World J Gastroenterol.

[37] Targher G, Bertolini L, Padovani R (2006). Increased prevalence of cardiovascular disease in type 2 diabetic patients with non-alcoholic fatty liver disease. Diabet Med.

[38] Targher G, Bertolini L, Padovani R (2007). Prevalence of nonalcoholic fatty liver disease and its association with cardiovascular disease among type 2 diabetic patients. Diabetes Care.

[39] Powell EE, Cooksley WGE, Hanson R, Searle J, Halliday JW, Powell W (1990). The natural history of nonalcoholic steatohepatitis: a follow-up study of forty-two patients for up to 21 years. Hepatology.

[40] Soderberg C, Stal P, Askling J (2010). Decreased survival of subjects with elevated liver function tests during a 28-year follow-up. Hepatology.

[41] Lazo M, Hernaez R, Bonekamp S (2011). Non-alcoholic fatty liver disease and mortality among US adults: prospective cohort study. BMJ.

[42] Kim D, Kim W, Kim HJ, Therneau TM (2013). Association between noninvasive fibrosis markers and mortality among adults with nonalcoholic fatty liver disease in the United States. Hepatology.

[43] D’Amico G, Garcia-Tsao G, Pagliaro L (2006). Natural history and prognostic indicators of survival in cirrhosis: a systematic review of 118 studies. J Hepatol.

[44] Kamath PS, Wiesner RH, Malinchoc M (2001). A model to predict survival in patients with end-stage liver disease. Hepatology.

