# Supplementary material for: γ-Glutamyltransferase, but not markers of hepatic fibrosis, is associated with cardiovascular disease in older people with type 2 diabetes mellitus: the Edinburgh Type 2 Diabetes Study
Source: Diabetologia. 2015 Mar 29;58(7):1484–93. doi: 10.1007/s00125-015-3575-y (PMC4473275; doi:10.1007/s00125-015-3575-y)
Supplement: Supplementary file 1 — (PDF 185 kb) [file 125_2015_3575_MOESM1_ESM.pdf]

ESM Table 1. Biomarkers of liver injury in all subjects with and without prevalent cardiovascular disease at baseline (values are mean (sd), median (IQR) or % (n)).

	CVD, yes N=370	CVD, no N=663	p value	CAD, yes N=317	CAD, no N=716	p value
ALT, U/L	41.9 (12.6)	43.7 (14.2)	0.048	42.1 (13.0)	43.5 (14.0)	0.164
AST, U/L	30.4 (10.5)	31.3 (10.0)	0.178	30.5 (11.0)	31.2 (9.8)	0.282
GGT, U/L	20.0 (13-37)	17.0 (10-29)	<0.001	20.0 (13-37)	17.0 (11-29)	<0.001
Steatosis, % yes[a]	54.1 (164)	57.5 (319)	0.350	51.2 (133)	58.5 (350)	0.051
CK18, U/L[a]	100.7 (79-141)	104.8 (77-137)	0.911	99.1 (74-138)	105.8 (78-138)	0.589
APRI	0.25 (0.19-0.35)	0.25 (0.20-0.33)	0.848	0.25 (0.19-0.36)	0.25 (0.20-0.33)	0.860
AST/ALT ratio	0.74 (0.2)	0.73 (0.2)	0.759	0.74 (0.2)	0.74 (0.2)	0.869
ELF score[b]	8.9 (0.8)	8.9 (0.9)	0.930	8.9 (0.8)	8.9 (0.9)	0.950
FIB4	1.39 (0.7)	1.34 (0.6)	0.164	1.42 (0.7)	1.33 (0.6)	0.060
NFS	-27.0 (2.5)	-27.4(2.6)	0.018	-26.9 (2.5)	-27.4 (2.6)	0.010
Platelets, $x10^9$/L	254.5 (72.9)	260.1 (67.8)	0.240	252.4 (74.3)	260.8 (67.4)	0.094

[a] Prevalent CVD n=303/858 prevalent CAD n=260/858; [b] Prevalent CVD n=234/679 prevalent CAD n=199/679.

ALT alanine aminotransferase; **APRI** aspartate aminotransferase to platelet ratio index; **AST** aspartate aminotransferase, **CAD** coronary artery disease; **CK18** cytokeratin-18; **CVD** cardiovascular disease; **ELF** Enhanced Liver Fibrosis; **FIB4** Fibrosis-4 score; **GGT** gammaglutamyl transferase; **NFS** NAFLD Fibrosis Score
