# Supplementary material for: γ-Glutamyltransferase, but not markers of hepatic fibrosis, is associated with cardiovascular disease in older people with type 2 diabetes mellitus: the Edinburgh Type 2 Diabetes Study
Source: Diabetologia. 2015 Mar 29;58(7):1484–93. doi: 10.1007/s00125-015-3575-y (PMC4473275; doi:10.1007/s00125-015-3575-y)
Supplement: Supplementary file 2 — (PDF 98 kb) [file 125_2015_3575_MOESM2_ESM.pdf]

ESM Table 2. Biomarkers of liver injury in subjects with and without incident cardiovascular disease (subset of subjects without prevalent CVD at baseline, values are mean (sd), median (IQR) or % (n)).

	CVD, yes N=35	CVD, no N=628	p value	CAD, yes N=19	CAD, no N=644	p value
ALT, U/L	46.0 (16.7)	43.5 (14.1)	0.266	48.2 (19.1)	43.5 (13.5)	0.218
AST, U/L	34.2 (13.0)	31.1 (9.7)	0.047	35.6 (14.6)	31.1 (9.7)	0.125
GGT, U/L	21.0 (10-37)	16.0 (10-27)	0.102	19.0 (9-56)	17.0 (10-28)	0.504
Steatosis, % yes[a]	50.0 (15)	57.8 (307)	0.450	43.8 (7)	57.8 (315)	0.309
CK18, U/L[a]	108.9 (85-146)	103.6 (76-137)	0.356	102.1 (84-176)	104.8 (77-137)	0.724
APRI	0.26 (0.21-0.34)	0.25 (0.20-0.33)	0.669	0.26 (0.20-0.40)	0.25 (0.20-0.33)	0.782
AST/ALT ratio	0.75 (0.2)	0.73 (0.2)	0.357	0.75 (0.1)	0.73 (0.2)	0.640
ELF score[b]	9.3 (0.8)	8.9 (0.9)	0.066	9.1 (0.7)	8.9 (0.9)	0.416
FIB4	1.39 (0.6)	1.33 (0.6)	0.494	1.42 (0.6)	1.33 (0.6)	0.417
NFS	-0.81 (1.1)	-0.72 (1.1)	0.600	-0.90 (1.0)	-0.72 (1.1)	0.440
Platelets, $x10^9/L$	266.9 (65.6)	259.8 (69.7)	0.502	269.7 (68.4)	259.9 (67.8)	0.460

Mean follow-up 4.4 years, except for CK18, ELF and steatosis where mean follow-up was 3.5 years.

[a] Incident CVD n=30/561 incident CAD n=16/561; [b] incident CVD n=24/444 incident CAD n=13/444.

ALT alanine aminotransferase; **APRI** aspartate aminotransferase to platelet ratio index; **AST** aspartate aminotransferase, **CAD** coronary artery disease; **CK18** cytokeratin-18; **CVD** cardiovascular disease; **ELF** Enhanced Liver Fibrosis; **FIB4** Fibrosis-4 score; **GGT** gammaglutamyl transferase; **NFS** NAFLD Fibrosis Score
