# Supplementary material for: γ-Glutamyltransferase, but not markers of hepatic fibrosis, is associated with cardiovascular disease in older people with type 2 diabetes mellitus: the Edinburgh Type 2 Diabetes Study
Source: Diabetologia. 2015 Mar 29;58(7):1484–93. doi: 10.1007/s00125-015-3575-y (PMC4473275; doi:10.1007/s00125-015-3575-y)
Supplement: Supplementary file 3 — (PDF 70 kb) [file 125_2015_3575_MOESM3_ESM.pdf]

ESM Table 3. Multivariable association between liver markers and incident cardiovascular disease events – contribution of included covariables. Values are hazard ratios (95%CI)

	All cardiovascular disease		Coronary artery disease	
	HR (95%CI)	p value	HR (95%CI)	p value
GGT, $\log_2$	1.25 (0.99, 1.59)	0.062	1.28 (0.99, 1.66)	0.064
+ Age	1.29 (1.05, 1.58)	0.016	1.30 (1.00, 0.68)	0.048
+ BP lowering medication	1.27 (1.03, 1.55)	0.024	1.28 (0.99, 1.65)	0.064
+ Lipid lowering medication	1.26 (1.03, 1.56)	0.028	1.28 (0.99, 1.67)	0.060
+ SIMD	1.27 (1.02, 1.58)	0.031	1.28 (0.97, 1.67)	0.080
+ Alcohol excess	1.25 (1.00, 1.54)	0.044	1.28 (0.96, 1.73)	0.099
+ Sex	1.24 (1.00, 1.53)	0.048	1.28 (0.98, 1.67)	0.068
+ Diabetes treatment type	1.23 (0.99, 1.54)	0.064	1.23 (0.93, 1.61)	0.145
+ Ever smoked	1.25 (0.98, 1.58)	0.069	1.28 (0.96, 1.71)	0.098
+ All	1.24 (0.97, 1.59)	0.086	1.33 (1.00, 1.78)	0.053

BP blood pressure; **GGT** gamma-gluyamyl transferase; **HR** hazard ratio; **SIMD** Scottish Index of Multiple Deprivation
