# Supplementary material for: γ-Glutamyltransferase, but not markers of hepatic fibrosis, is associated with cardiovascular disease in older people with type 2 diabetes mellitus: the Edinburgh Type 2 Diabetes Study
Source: Diabetologia. 2015 Mar 29;58(7):1484–93. doi: 10.1007/s00125-015-3575-y (PMC4473275; doi:10.1007/s00125-015-3575-y)
Supplement: Supplementary file 4 — (PDF 165 kb) [file 125_2015_3575_MOESM4_ESM.pdf]

ESM Table 4. Multivariable association between liver markers and incident cardiovascular disease events (all subjects). Values are hazard ratios (95%CI)

	Model 1	p value	Model 2	p value	Model 3	p value
ALT, U/L	0.99 (0.98,1.01)	0.379	0.99 (0.98,1.01)	0.262	0.99 (0.97,1.00)	0.140
AST, U/L	1.00 (0.99,1.02)	0.674	1.00 (0.98,1.02)	0.888	1.00 (0.98,1.02)	0.794
GGT, $\log_2$ [a]	1.28 (1.10,1.49)	0.001	1.26 (1.08,1.47)	0.003	1.18 (1.00,1.39)	0.045
Steatosis, % yes[b]	1.24 (0.75,2.04)	0.399	1.32 (0.80,2.17)	0.282	1.31 (0.78,2.20)	0.307
CK18, $\log_2$ [a, b]	1.15 (0.84,1.56)	0.380	1.20 (0.88,1.64)	0.254	1.07 (0.78,1.46)	0.687
APRI, $\log_2$ [a]	0.97 (0.69,1.36)	0.873	0.81 (0.56,1.17)	0.263	0.78 (0.54,1.13)	0.193
AST:ALT ratio	2.90 (0.92,9.16)	0.069	2.67 (0.86,8.33)	0.090	2.85 (0.90,8.98)	0.074
ELF score[c]	1.20 (0.98,1.48)	0.082	1.23 (0.97,1.56)	0.095	1.15 (0.88,1.50)	0.304
FIB4	1.15 (0.82,1.62)	0.421	0.94 (0.63,1.39)	0.747	0.91 (0.61,1.34)	0.621
NFS	0.97 (0.90, 1.06)	0.542	0.98 (0.90,1.07)	0.642	0.96 (0.88,1.04)	0.320
Platelets, $x10^9$/L	1.00 (1.00,1.00)	0.596	1.00 (1.00,1.01)	0.071	1.00 (1.00,1.01)	0.101

[a] APRI, CK18 and GGT analysed on the Log_2 scale for linearization, therefore odds ratios relate to a doubling of the marker; [b] Incident CVD n=30/561; [c] incident CVD n=24/444.

Model 1 – Unadjusted; Model 2 - Adjusted for age and sex; Model 3 - Adjusted for age, sex, duration of diabetes, treatment of diabetes, lipid lowering drugs, blood pressure lowering drugs, deprivation (Scottish Index of Multiple Deprivation quintile), smoking status, excess alcohol consumption, body mass index,

systolic blood pressure, diastolic blood pressure, HbA1c, HDL cholesterol, total cholesterol and estimated glomerular filtration rate. Incident analysis additionally adjusted for prevalence cardiovascular disease at baseline.

ALT alanine aminotransferase; **APRI** aspartate aminotransferase to platelet ratio index; **AST** aspartate aminotransferase; **CK18** cytokeratin-18; **ELF** Enhanced Liver Fibrosis; **FIB4** Fibrosis-4 score; **GGT** gammaglutamyl transferase; **NFS** NAFLD Fibrosis Score
