# Supplementary material for: γ-Glutamyltransferase, but not markers of hepatic fibrosis, is associated with cardiovascular disease in older people with type 2 diabetes mellitus: the Edinburgh Type 2 Diabetes Study
Source: Diabetologia. 2015 Mar 29;58(7):1484–93. doi: 10.1007/s00125-015-3575-y (PMC4473275; doi:10.1007/s00125-015-3575-y)
Supplement: Supplementary file 5 — (PDF 88 kb) [file 125_2015_3575_MOESM5_ESM.pdf]

ESM Table 5. Multivariable association between liver markers and incident coronary artery disease events (all subjects). Values are hazard ratios (95%CI)

	Model 1	p value	Model 2	p value	Model 3	p value
ALT, U/L	1.00 (0.98,1.02)	0.976	1.00 (0.98,1.02)	0.911	1.00 (0.98,1.02)	0.742
AST, U/L	1.02 (1.00,1.04)	0.148	1.02 (0.99,1.04)	0.151	1.01 (0.99,1.03)	0.316
GGT, log_2[a]	1.31 (1.10,1.58)	0.003	1.33 (1.11,1.59)	0.002	1.24 (1.02,1.51)	0.032
Steatosis, % yes[b]	1.62 (0.86,3.05)	0.133	1.70 (0.90,3.20)	0.103	1.12 (0.82,3.04)	0.175
CK18, log_2[a, b]	1.15 (0.81,1.65)	0.440	1.20 (0.83,1.73)	0.328	1.08 (0.74,1.57)	0.687
APRI, log_2[a]	1.06 (0.70,1.60)	0.775	0.98 (0.63,1.53)	0.932	0.93 (0.60,1.45)	0.751
AST:ALT ratio	3.95 (1.03,15.17)	0.045	3.39 (0.87,13.28)	0.080	3.72 (0.96,14.53)	0.058
ELF score[c]	1.18 (0.89,1.55)	0.251	1.13 (0.81,1.57)	0.474	1.019 (0.68,1.49)	0.963
FIB4	1.31 (0.89,1.92)	0.167	1.16 (0.75,1.79)	0.504	1.11 (0.72,1.71)	0.628
NFS	1.00 (0.90,1.10)	0.934	0.99 (0.89,1.10)	0.859	0.97 (0.87,1.07)	0.535
Platelets, $x10^9$/L	1.00 (1.00,1.0)	0.359	1.00 (1.00,1.01)	0.114	1.00 (1.00,1.01)	0.132

[a] APRI, CK18 and GGT analysed on the Log_2 scale for linearization, therefore odds ratios relate to a doubling of the marker; [b] incident CAD n=16/561; [c] incident CAD n=13/444.

Model 1 – Unadjusted; Model 2 - Adjusted for age and sex; Model 3 - Adjusted for age, sex, duration of diabetes, treatment of diabetes, lipid lowering drugs, blood pressure lowering drugs, deprivation (Scottish Index of Multiple Deprivation quintile), smoking status, excess alcohol consumption, body mass index,

systolic blood pressure, diastolic blood pressure, HbA1c, HDL cholesterol, total cholesterol and estimated glomerular filtration rate. Incident analysis additionally adjusted for prevalence cardiovascular disease at baseline.

ALT alanine aminotransferase; **APRI** aspartate aminotransferase to platelet ratio index; **AST** aspartate aminotransferase; **CK18** cytokeratin-18; **ELF** Enhanced Liver Fibrosis; **FIB4** Fibrosis-4 score; **GGT** gammaglutamyl transferase; **NFS** NAFLD Fibrosis Score
