# Supplementary material for: γ-Glutamyltransferase, but not markers of hepatic fibrosis, is associated with cardiovascular disease in older people with type 2 diabetes mellitus: the Edinburgh Type 2 Diabetes Study
Source: Diabetologia. 2015 Mar 29;58(7):1484–93. doi: 10.1007/s00125-015-3575-y (PMC4473275; doi:10.1007/s00125-015-3575-y)
Supplement: Supplementary file 6 — (PDF 162 kb) [file 125_2015_3575_MOESM6_ESM.pdf]

ESM Table 6. Multivariable association between liver markers and any incident cardiovascular disease events (subjects with NAFL). Values are hazard ratios (95%CI)

	Model 1	p value	Model 2	p value	Model 3	p value
ALT, U/L	0.99 (0.97,1.02)	0.446	0.98 (0.968,1.01)	0.236	0.98 (0.96,1.01)	0.219
AST, U/L	1.01 (0.97,1.04)	0.800	1.00 (0.97,1.04)	0.851	1.00 (0.96,1.04)	0.860
GGT, log_2[a]	1.58 (1.15,2.17)	0.005	1.57 (1.13,2.18)	0.007	1.56 (1.08,2.28)	0.019
CK18, log_2[a]	0.91 (0.54,1.52)	0.711	0.91 (0.55,1.52)	0.718	0.98 (0.58,1.66)	0.944
APRI, log_2[a]	0.94 (0.52,1.70)	0.840	0.80 (0.43,1.51)	0.495	0.69 (0.36,1.31)	0.691
AST:ALT ratio	8.14 (0.77,85.48)	0.081	26.62 (2.03,349.29)	0.012	20.10 (1.36,296.91)	0.029
ELF score	1.10 (0.80,1.50)	0.567	1.19 (0.86,1.66)	0.294	1.20 (0.84,1. 07)	0.317
FIB4	1.06 (0.47,2.35)	0.895	0.92 (0.39,2.19)	0.854	0.70 (0.28,1.73)	0.436
NFS	0.96 (0.83,1.11)	0.564	0.98 (0.84,1.14)	0.784	0.97 (0.84,1.12)	0.682
Platelets, $x10^9$/L	1.00 (1.00,1.01)	0.479	1.00 (1.00,1.01)	0.145	1.01 (1.00,1.01)	0.068

ALT, AST, GGT, APRI, AST:ALT ratio, FIB4, NFS and platelets total n=319 incident CVD n=38; CK18 total n=295 incident CVD n=29; ELF total n=231 incident CVD n=26.

[a] APRI, CK18 and GGT analysed on the Log_2 scale for linearization, therefore odds ratios relate to a doubling of the marker.

Model 1 – Unadjusted; Model 2 - Adjusted for age and sex; Model 3 - Adjusted for age, sex, duration of diabetes, treatment of diabetes, lipid lowering drugs, blood pressure lowering drugs, deprivation (Scottish Index of Multiple Deprivation quintile), smoking status, excess alcohol consumption, body mass index,

systolic blood pressure, diastolic blood pressure, HbA1c, HDL cholesterol, total cholesterol and estimated glomerular filtration rate. Incident analysis additionally adjusted for prevalence cardiovascular disease at baseline.

ALT alanine aminotransferase; **APRI** aspartate aminotransferase to platelet ratio index; **AST** aspartate aminotransferase; **CK18** cytokeratin-18; **ELF** Enhanced Liver Fibrosis; **FIB4** Fibrosis-4 score; **GGT** gammaglutamyl transferase; **NFS** NAFLD Fibrosis Score
